# Altered performance in a rat gambling task after acute and repeated alcohol exposure

**DOI:** 10.1007/s00213-015-4020-0

**Published:** 2015-07-30

**Authors:** Marcia Spoelder, Heidi M. B. Lesscher, Peter Hesseling, Annemarie M. Baars, José G. Lozeman-van t Klooster, Rob Mijnsbergen, Louk J. M. J. Vanderschuren

**Affiliations:** Department of Animals in Science and Society, Division of Behavioural Neuroscience, Faculty of Veterinary Medicine, Utrecht University, Yalelaan 2, 3584 CM Utrecht, The Netherlands; Department of Translational Neuroscience, Brain Center Rudolf Magnus, University Medical Center Utrecht, Utrecht, The Netherlands

**Keywords:** Alcohol, Disinhibition, Decision making, Punishment, Impulsivity

## Abstract

**Rationale:**

A bidirectional relationship between alcohol use disorder (AUD) and deficits in impulse control and decision making has been suggested. However, the mechanisms by which neurocognitive impairments predispose to, or result from AUD remain incompletely understood.

**Objectives:**

The aim of this study is to gain more insight in the effects of alcohol exposure on decision making and impulse control. We used two modified versions of the rat gambling task (rGT) that differ in the net gain and the punishment magnitude associated with the different response options.

**Methods:**

In experiment 1, we assessed the effects of acute alcohol treatment (0–0.8 g/kg) on rGT performance. In experiment 2, we determined the effects of alcohol on rGT acquisition (15 sessions, 0.6 g/kg). Next, these animals were challenged with alcohol (0–1.0 g/kg) prior to rGT sessions.

**Results:**

Acute alcohol treatment suppressed baseline performance in both rGT versions but only modestly altered decision making. Treatment with alcohol during acquisition increased risky choices in the rGT version that involved larger punishment and blunted the reduction in win-shift behavior during acquisition in both rGT versions. Moreover, rats treated with alcohol during acquisition showed an increase in premature and perseverative responding upon subsequent alcohol challenges (0–1.0 g/kg) and were less sensitive to the behavioral suppressant effects of alcohol.

**Conclusions:**

Our results show that repeated alcohol exposure alters decision making during rGT acquisition and reduces the ability to adjust choice behavior on the basis of feedback. In addition, repeated alcohol exposure unmasks its behavioral disinhibitory effects in the rGT. Impaired responsiveness to choice feedback and behavioral disinhibition may contribute to the development of AUD.

## Introduction

Alcohol is one of the most widely abused substances worldwide, and the problems associated with alcohol use disorder (AUD) pose a major burden to our society (World Health Organization 2011). Estimates are that 76 million people worldwide suffer from AUD (United Nations Office on Drugs and Crime 2012), and AUD is among the most financially costly of all major neuropsychiatric disorders (Effertz and Mann 2013). An important characteristic of AUD is compromised impulse control and decision making, which has been implicated in the development and maintenance of the disorder, as well as the likelihood of relapse (Garavan and Stout 2005; De Wit 2009; Rogers et al. 2010; Dalley et al. 2011; Fineberg et al. 2014). Thus, personality traits such as impulsivity, i.e., the tendency to act without consideration of possible consequences, and suboptimal decision making are alleged risk factors for AUD (Dom et al. 2006; Johnson et al. 2008; de Wit 2009; Goudriaan et al. 2011; King et al. 2011; Dalley et al. 2011). Conversely, AUD patients show maladaptive decision making and reduced impulse control (Bechara et al. 2001; Salgado et al. 2009; Kim et al. 2011; MacKillop et al. 2011; Tomassini et al. 2012; Voon et al. 2014). Hence, impaired decision making and impulsive behavior appear both to predispose to, and be a consequence of AUD, but cause and effect in the relationship between neurocognitive impairments and AUD can be difficult to disentangle in human studies (Ersche et al. 2013). Preclinical studies that afford more control over experimental subjects and conditions may therefore aid to understand the relationship between alcohol use, decision making, and impulsive behavior (Tomie et al. 1998; Mitchell et al. 2011; Walker et al. 2011; Irimia et al. 2013), to contribute to the prevention and treatment of AUD (Marhe et al. 2014).

The Iowa gambling task (IGT) is widely used to assess decision making in humans. This task combines several factors that guide everyday decision making, including probabilistic reward and punishment and the necessity to exert behavioral control in order to maximize long-term gains (Bechara et al. 1994). During task acquisition, when the contingencies of the response options are being learned, choice behavior changes from exploratory sampling to more exploitative advantageous decision making. However, despite the hypothesized relationship between AUD and impaired decision making, studies on the effects of acute exposure to alcohol on decision making have not been conclusive. Acute alcohol exposure studies in humans have reported an increase in disadvantageous choices (Lane et al. 2004; George et al. 2005), but also unaltered decision making (Ramaekers and Kuypers 2006). In a rodent version of the IGT (rat gambling task (rGT); Zeeb et al. 2009) and a comparable risky decision making task (Simon et al. 2009), acute alcohol exposure did not change decision making (Mitchell et al. 2011; Peña-Oliver et al. 2014). Importantly, in these rodent models, task acquisition takes several weeks of training, whereas in human studies, acquisition and performance of the IGT is usually examined in a single session. Hence, to investigate the effects of alcohol under choice uncertainty in a rodent model, alcohol should preferably be administered during task acquisition when the animals are learning the response-outcome associations.

Here, we aimed to gain further insight into the effects of alcohol exposure on decision making by examining the effects of single and repeated alcohol treatment in two modified versions of the rGT (Zeeb et al. 2009). The animals were offered three choice options, labeled safe, optimal, and risky, whereby the task versions were set up such that the choice contingencies differ in the magnitude and probability of reward delivery and punishment. By comparing the effects of alcohol on choice behavior in the two rGT versions, we investigated whether alcohol affects decision making by changing the responsiveness to reward or punishment or if alcohol evokes risky behavior. First, we assessed the acute effects of alcohol on stable choice behavior in the two rGT versions. Next, the effects of repeated administration of alcohol on task acquisition were determined, followed by alcohol challenge sessions after choice behavior had stabilized. Based on previous findings (Mitchell et al. 2011; Peña-Oliver et al. 2014), we hypothesized that acute alcohol administration has limited effects on decision making in animals that show stable choice behavior, whereas repeated alcohol administration during task acquisition results in disadvantageous decision making, similar to what has been shown in human studies (Lane et al. 2004; George et al. 2005). In addition, we also assessed the effects of alcohol on other behavioral parameters, i.e., choice latencies, omissions, perseverative responses, and responses during the intertrial interval (ITI), i.e., a premature response, which is considered to be a measure of motor impulsivity (Robbins 2002; Pattij and Vanderschuren 2008). Previous studies in humans and rodents have described that alcohol exposure results in impaired inhibitory control, especially after repeated binge-like alcohol exposure (Easdon and Vogel-Sprott 2000; Marczinski et al. 2007; Irimia et al. 2013; Sanchez-Roige et al. 2014). Hence, we expected to find differential effects of acute and repeated alcohol treatment on these behavioral parameters, especially on motor impulsivity (Bizarro et al. 2003; Peña-Oliver et al. 2009; Walker et al. 2011; Semenova 2012; Irimia et al. 2013).

## Materials and methods

### Animals

Male Lister Hooded rats (Charles River, Germany) weighing 220–250 g at the start of experimental training were used. The rats were housed in groups of 3–4 rats/cage under controlled temperature and humidity conditions and a reversed light/dark cycle (lights on 7.00 AM –lights off 7.00 PM) with ad libitum access to water and chow. After 2-week acclimatization to the housing conditions, the rats were gradually restricted to 5 g chow/100 g body weight/day, which maintained them at 90 % of their free-feeding weight. Body weights were monitored weekly, and the animals were briefly restrained during the weighing procedure, to habituate them to the injection procedure. All experiments were approved by the Animal Ethics Committee of Utrecht University and were conducted in agreement with Dutch Laws (Wet op de Dierproeven 1996) and European regulations (Guideline 86/609/EEC).

### Apparatus

Training and testing was conducted in operant conditioning chambers, illuminated by a white house light, in ventilated sound-attenuating cubicles (Med Associates, St. Albans, VT, USA). Chambers were equipped with an array of five holes in a curved wall, each with an infrared detector and a stimulus light. Sucrose pellets (TestDiet, UK) could be delivered at the opposite wall via a dispenser. The apparatus was controlled using MEDPC software version 1.17 (Med Associates).

### Behavioral procedures

During training, the animals were required to make a nose-poke in the illuminated aperture to obtain sucrose pellets, as described previously (Zeeb et al. 2009; Baarendse et al. 2013). Subsequently, the rats were trained in one of two rGT versions (GT1 or GT2), both with three choices, termed safe, optimal, and risky (Table 1). Since choice for one of the response options (choice P3) in the 4 choice rGT has been shown not to change over time (Zeeb et al. 2009; Baarendse et al. 2013), we modified the task by excluding this response option. The rGT versions were designed to have one optimal choice and two suboptimal choice options (i.e., safe and risky). Feedback about the contingencies of the three different choices was provided in the form of the number of sucrose pellets received, the probability of receiving the reward, and punishment magnitude. Punishment consisted of a time-out period, which reduced the available session time and resulted in a lower net gain. In GT1, the net gain was different between all three choices, the gain being highest for the optimal choice > safe choice (72 % of optimal) > risky choice (24 % of optimal). In order to investigate the effect of reward size and probability in the risky choice separately from the lower net gain, we designed GT2 such that the punishment time out of the risky choice was relatively short, making the net gain of the risky choice (66 % of optimal) more comparable to the net gain of the safe choice (76 % of optimal). Thus, in both rGT versions, the risky choice provides the animal with a high number of sucrose pellets in case the animal is rewarded. However, the negative consequence, i.e., the punishment time-out, is higher in GT1 compared to GT2, resulting in a threefold lower net gain in GT1. Moreover, in GT2, the safe choice consists of a nonprobabilistic choice (one pellet with 100 % reward probability) resulting in a larger difference in probability between the safe and optimal choice. The spatial location of the three choices was counterbalanced across subjects in the two rGT versions and remained the same for each animal over the course of the experiment. The middle three response holes of the five-hole array were used. The order of the options from left to right in version A was risky–optimal–safe and in version B safe–risky–optimal. The animals were tested for 30 min per session, 5–6 days/week.

**Table 1:** Choice contingencies of the safe, optimal, or risky choice in rGT version 1 (GT1) and rGT version 2 (GT2)

rGT	Choice	No. of pellets	Chance %	Punishment time-out (s)	Theoretical gain	Ratio of long-term gain
GT1	Safe	1	90	5	294	0.72
	Optimal	2	80	10	411	–
	Risky	4	40	40	99	0.24
GT2	Safe	1	100	0	360	0.76
	Optimal	3	70	10	473	–
	Risky	6	33	10	310	0.66

No. of pellets: the number of sucrose pellets the animal receives when rewarded; chance %: the chance to receive a reward; duration of the punishment time-out; theoretical gain: number of pellets that would be obtained if this option was chosen exclusively, which provides an objective value for each response option. Theoretical gain is calculated as ((1800 s of session duration / ((5 s ITI + (chance of punishment × punishment time-out in seconds)) × (chance of reward × number or pellets)). Ratio of long-term gain: number of sucrose pellets, which could be theoretically obtained from that response option, divided by the theoretical number of sucrose pellets of the optimal choice

The task design and trial structure were as previously described (Zeeb et al. 2009; Baarendse et al. 2013). Briefly, a trial started with a 5-s ITI, followed by illumination of one (during forced-choice sessions) or three (during free choice sessions) stimulus lights for 10 s. A response in an illuminated hole turned off the stimulus light(s), and led to either reward or punishment. During forced-choice sessions, only one stimulus light was illuminated in a pseudorandom order to ensure that all animals had equal experience with the contingencies of the three choice options. A nose poke response in a nonilluminated aperture (i.e., incorrect response), a failure to respond within 10 s (i.e., omission), or a premature response resulted in a 5-s time-out period, signaled by illumination of the house light. Nose poke responses in the stimulus holes during punishment were scored as perseverative responses, but these had no scheduled consequences.

#### Experiments

Experiment 1 was designed to compare the development of choice behavior over sessions in the two rGT versions and to assess the effects of acute alcohol treatment on stable responding in both rGT versions. The rats were first tested for five free choice sessions, to investigate if the animals would develop a choice preference by spontaneously sampling the choices. As we noted that not all rats explored all three choices, we subsequently introduced five forced-choice sessions to ensure that all animals had equal experience with the contingencies of the three options before they were tested for another 21 free-choice sessions. Subsequently, the animals were treated with five doses of alcohol (0–0.8 g/kg) prior to rGT testing.

In experiment 2, we determined the effect of treatment with a moderate dose of alcohol (0.6 g/kg) on the acquisition of GT1 and GT2. The dose of 0.6 g/kg was chosen because it was the lowest dose that showed significant effects on behavior in the rGT without causing profound depressant effects in experiment 1 (see Table 2). This experiment started with five forced-choice sessions to ensure that the effects of alcohol or vehicle on the development of choice behavior were not skewed by rats not knowing all three choice contingencies. Subsequently, the animals were subjected to 15 free-choice sessions prior to which they received alcohol or vehicle injections. Thereafter, the animals were left undisturbed for 2 days to ensure complete washout of the alcohol. Subsequently, the animals were trained for ten more sessions without any treatment. Finally, 3 days after the last nontreatment session, all animals were challenged with five doses of alcohol (0–1.0 g/kg).

**Table 2:** Effects of alcohol on behavior in the rGT

Variable	Dose effect	Vehicle	0.2 g/kg	0.4 g/kg	0.6 g/kg	0.8 g/kg
Number of choices	*F*_(3,100)_ = 6.362, *p* < 0.05	65.75 ± 3.29	63.00 ± 2.85	60.34 ± 3.00	61.22 ± 2.67	50.13* ± 4.13
Premature responses	*F*_(4,120)_ = 25.349, *p* < 0.05	18.47 ± 1.39	17.53 ± 1.46	15.53* ± 1.19	9.38* ± 1.21	6.28* ± 0.92
PersevP	*F*_(4,120)_ = 15.017, *p* < 0.05	21.28 ± 2.03	22.00 ± 2.46	17.44 ± 2.55	15.16* ± 2.15	9.28* ± 1.60
Omissions	*F*_(3,93)_ = 9.495, *p* < 0.05	24.69 ± 4.01	26.97 ± 3.43	26.50 ± 3.77	33.72* ± 3.38	46.06* ± 5.03
Choice latency (sec)	*F*_(4,120)_ = 10.998, *p* < 0.05	3.53 ± 0.19	3.50 ± 0.19	3.59 ± 0.17	4.01* ± 0.18	4.16* ± 0.13
Collect latency (sec)	*F*_(4,120)_ = 3.553, *p* < 0.05	2.12 ± 0.15	2.48 ± 0.24	2.69 ± 0.31	2.83* ± 0.27	2.82* ± 0.23

Data are presented as means ± SEM. *F* values represent the main effect of alcohol dose (repeated-measures ANOVA). Post hoc analyses were performed by paired *t* tests, comparing alcohol doses to vehicle. Data from both rGT versions were pooled because the rGT version did not interact with the effects of alcohol (i.e., there were no GT × dose interactions). *Different from vehicle, *p* < 0.05

*PersevP* perseverative responses during a punishment trial

### Drugs

Alcohol (99.5 %, Klinipath, The Netherlands) was diluted with saline to a concentration of 10 % alcohol (*v*/*v*). Injection volumes were adjusted to the body weight and the required dose of alcohol. The alcohol solutions were preheated to 32 °C by placing the syringes on a heating pad to prevent possible decreases in body temperature after injection of substantial volumes, particularly at the highest alcohol doses. Vehicle (i.e., saline) injection volumes were equivalent to the volume required for an injection of the 0.6 g/kg alcohol dose. Drug solutions were freshly prepared daily and administered intraperitoneally (IP) 15 min prior to behavioral testing. Prior to injections, the rats were habituated two times to the injection procedure. The different alcohol challenge doses were administered according to a Latin square design with a three day cycle for each dose, i.e., a baseline session, followed by the alcohol treatment session and a washout day during which the animals remained in their home-cage. During the 15 acquisition sessions with alcohol or vehicle treatment in experiment 2, all animals received injections prior to rGT training on Monday, Tuesday, Thursday, and Friday. The animals remained in their home-cage on Wednesdays and during the weekend in order to minimize irritation of the peritoneal cavity which is potentially caused by repeated injection of the alcohol solution.

### Blood alcohol levels

In a separate group of animals, we determined blood alcohol levels (BALs) after an IP injection of 0.6 and 1.2 g/kg alcohol. Thirty minutes after injection, blood samples were collected from the lateral tail vein in EDTA-coated capillary tubes (Sarstedt, Numbrecht, Germany) and immediately stored on ice. In addition, to explore the metabolism of alcohol over time, animals were treated with 0.6 g/kg for blood sampling at 5, 10, 15, 30, 60, and 120 min after injection. Blood samples were spun at 3000 rpm for 20 min (at 4 °C) and plasma was stored at −20 °C until blood alcohol analysis. BALs (mg/dl) were determined using an NAD-ADH reagent kit (Sigma-Aldrich, Schnelldorf, Germany) and a standard curve for quantitation.

### Data analysis

All statistical analyses were conducted using SPSS 20.0 for Windows. Statistical analyses were performed using one-, two-, and three-way repeated-measures ANOVAs with choice, session, and dose as within-subject variables and treatment group (alcohol or vehicle) and/or rGT version (GT1 and GT2) as between-subject variables. Trial-by-trial analysis was performed to assess the shifts in choice behavior between subsequent trials. Depending on whether the animal received a reward or a punishment, it can make the same choice on the subsequent trial or shift toward another choice option, resulting in four different possibilities (i.e., win-stay, win-shift, lose-stay, lose-shift) per choice option (safe, optimal, or risky), resulting in 12 different possibilities in total. Because not all of these possibilities occurred in each session for each individual animal, the data of the trial-by-trial analyses per choice were averaged over five sessions to obtain reliable data points for each animal, calculated as a percentage. For example, lose-shift behavior after a risky choice was calculated by dividing the number of shifts upon a loss on the risky choice by the total number of losses on the risky choice, multiplied by 100. In addition, we analyzed the percentage of shifts toward another choice option after rewarded and punished trials, regardless of which option was chosen. The total percentage of win-shifts was calculated by dividing the number of win-shifts by the total number of wins during the session, multiplied by 100; the total percentage of lose-shifts was calculated analogously. The data was tested for normality with a Kolmogorov-Smirnov test. When data were not normally distributed, data was square root transformed for count data (e.g., premature responses) and log-transformed for latency data, which resulted in normal distribution of the data in all cases. Choice behavior data and the trial-by-trial data, expressed as percentages, were arcsine-transformed. Mauchly’s test of sphericity was used to test if variances of the differences between treatment levels were equal. If the assumption of sphericity was violated, degrees of freedom were corrected using Huynh-Feldt estimates of sphericity to more conservative values. Corrected degrees of freedom are presented rounded to the nearest integer. Paired *t* tests were used as post hoc analyses to compare a drug dose with vehicle. Behavioral parameters of both rGT versions were pooled in case the rGT version did not interact with alcohol treatment effects (i.e., absence of rGT version × dose / session interaction). The threshold for statistical significance was set at *p* < 0.05. All data are presented as mean ± SEM. Graphs were made using GraphPad Prism 6.

## Results

### Choice behavior in GT1 and GT2

**Fig. 1:**
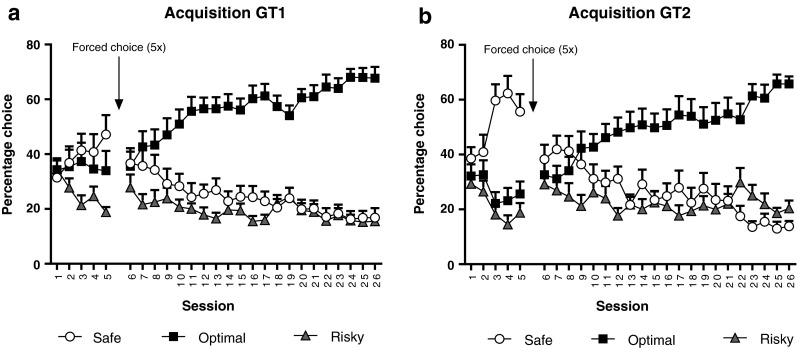
Acquisition of choice behavior in GT1 (**a**) and GT2 (**b**). Choice behavior during the first five free-choice sessions differed between the two gambling tasks, in that rats showed a higher preference for the safe choice in GT2. Moreover, while animals in GT1 preferred the safe and optimal choice above the risky choice, animals in GT2 preferred the safe choice above the optimal and risky choice. Following five forced-choice sessions, rats in both rGT versions developed a preference for the optimal choice, which became more pronounced with increased training. Data are shown as the mean percentage choice + SEM

Over the course of the first five free-choice sessions in experiment 1, animals changed their choice behavior in a version-dependent manner (*F*_(8,165) GT × choice × session_ = 2.33, *p* < 0.05) (Fig. 1). Animals in GT1 (Fig. 1a) initially preferred both the safe and optimal choice above the risky choice (*p* < 0.05), whereas animals in GT2 (Fig. 1b) preferred the safe choice above the optimal and risky choice (*p* < 0.05). Analyses of the 21 free-choice sessions that followed the five forced-choice sessions indicated that animals developed a preference for the optimal choice (*F*_(16,465) choice × session_ = 9.50, *p* < 0.05), which can be expected on the basis of the theoretical gain that is highest for the optimal choice. The overall choice pattern in these 21 free-choice sessions did not differ between the two rGT versions (*F*_(16,465) choice × GT × session_ = 0.64, NS) (Fig. 1).

### Blood alcohol levels

**Fig. 2:**
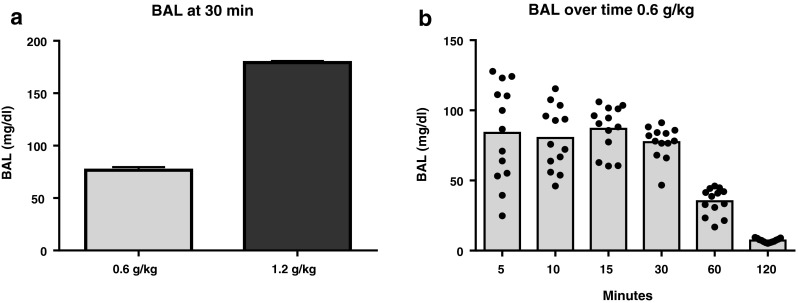
Blood alcohol level (BAL) after an IP alcohol injection. The BAL was assessed in a separate group of animals at 30 min after IP injection of 0.6 and 1.2 g/kg alcohol (**a**). Investigation of the BAL over time after an injection with 0.6 g/kg alcohol showed maximal BAL with least variation at 15–30 min postinjection (**b**). Data are shown as the mean + SEM (**a**) or as mean and individual data points (**b**)

Thirty minutes after injection of 0.6 or 1.2 g/kg alcohol, BAL amounted to 77 ± 3 and 179 ± 1 mg/dl, respectively, which is near and above the legal alcohol limit of 80 mg/dl (Fig. 2a). Investigation of the BAL over time after injection of 0.6 g/kg alcohol, showed maximal BAL with least variation between animals at 15–30 min postinjection. BAL approached zero after 120 min (Fig. 2b).

### Acute effects of alcohol on rGT performance

**Fig. 3:**
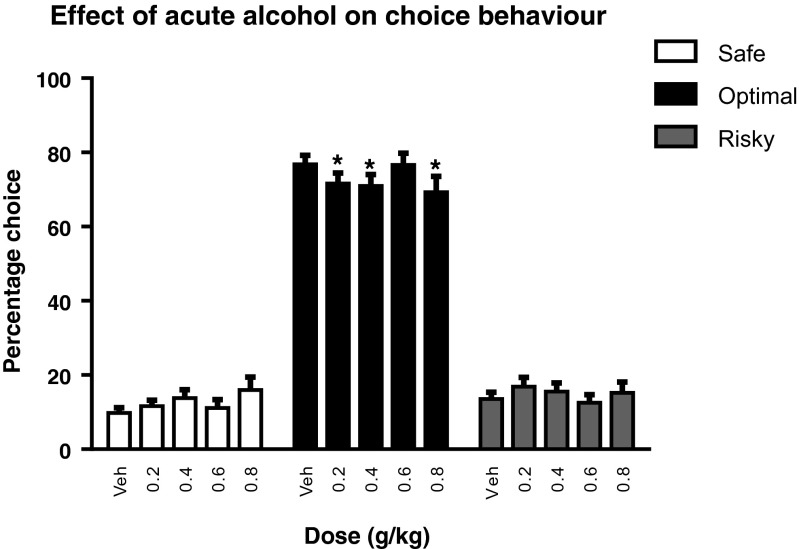
The effect of acute alcohol treatment on stable choice behavior in the rGT (experiment 1). Alcohol significantly reduced the percentage of optimal choices. This effect of alcohol was independent of GT version. Hence, the data from both rGT versions were collapsed. Data are shown as the mean percentage choice + SEM. *Different from vehicle treatment (post hoc paired *t* test, *p* < 0.05)

Alcohol changed choice behavior (*F*_(6,173) choice × dose_ = 2.33, *p* < 0.05) independent of rGT version (*F*_(6,173) choice × dose × GT_ = 0.95, NS) (Fig. 3). Alcohol reduced the percentage of optimal choices (*F*_(3,93) dose_ = 3.10, *p* < 0.05) and post hoc analyses revealed a decrease in optimal choices at the doses of 0.2, 0.4, and 0.8 g/kg (*p* < 0.05). Alcohol did not affect the percentage of safe choices (*F*_(2,75) dose_ = 1.93, NS), nor did it alter the percentage of risky choices (*F*_(4,124) dose_ = 1.82, NS) (Fig. 3). Treatment with alcohol reduced the number of choices (0.8 g/kg), premature (0.4–0.8 g/kg) and perseverative responses (0.6–0.8 g/kg) and increased the number of omissions (0.6–0.8 g/kg), choice latencies (0.6–0.8 g/kg), and collect latencies (0.6–0.8 g/kg) (Table 2).

### Effect of alcohol on rGT acquisition

**Fig. 4:**
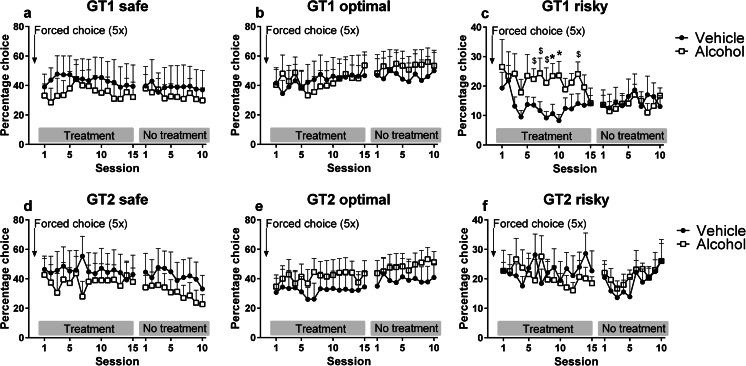
The effects of repeated alcohol (0.6 g/kg) or vehicle administration on the acquisition of choice behavior in the rGT, followed by ten sessions without treatment. Repeated alcohol administration during rGT acquisition increased risky choices in GT1 (**c**). Data are shown as the mean percentage choice + SEM. *Different from vehicle-treated animals (post hoc Student’s *t* test, *p* < 0.05), $ *p* < 0.062 compared to vehicle-treated rats

In experiment 2, rats were repeatedly treated with alcohol (0.6 g/kg) or vehicle prior to the first 15 free-choice acquisition sessions in the rGT. Analysis of the choice behavior in these sessions revealed a significant interaction between treatment, rGT version, and session (*F*_(11,294) treatment × GT × session_ = 2.00, *p* < 0.05) (Fig. 4). Separate analyses per rGT version showed an effect of alcohol on choice pattern in GT1 (Fig. 4a–c) (*F*_(7,100) treatment × session_ = 2.87, *p* < 0.05), but not in GT2 (Fig. 4d–f) (*F*_(14,196) treatment × session_ = 0.75, NS). To further explore the effects of alcohol on choice behavior in GT1, we performed analyses per choice and found that alcohol-treated animals showed a higher percentage of risky choices (*F*_(1,14) treatment_ = 5.74, *p* < 0.05), but made a comparable percentage of safe (*F*_(1,14) treatment_ = 0.25, NS) and optimal choices (*F*_(1,14) treatment_ = 0.01, NS) compared to vehicle-treated animals. Post hoc analyses of the risky choice data showed that alcohol-treated animals made more risky choices during sessions 6–10 of alcohol treatment in comparison to vehicle-treated animals (Fig. 4c).

Subsequently, to assess the persistence of the effects of alcohol treatment, choice behavior was determined for another ten sessions after cessation of alcohol treatment. In contrast to the treatment period, we did not find an interaction between treatment, rGT version and session on choice behavior during this stage of the experiment (F_(8,364) treatment × GT × session_ = 1.17, NS) (Fig. 4). Importantly, in these ten sessions without treatment, there was a significant interaction between choice and session (*F*_(13,592) choice × session_ = 3.22, *p* < 0.05), which was independent of treatment (*F*_(13,592) choice × session × treatment_ = 0.71, NS) or rGT version (*F*_(13,592) choice × session × GT_ = 0.86, NS). Subsequent analyses per choice indicated that the percentage of safe choices decreased over sessions (*F*_(7,205) session_ = 4.18, *p* < 0.05), whereas the percentage of optimal choices did not change (*F*_(7,198) session_ = 1.14, NS). The percentage of risky choices changed over sessions (*F*_(8,224) session_ = 2.52, *p* < 0.05), but post hoc analysis revealed no differences with the first posttreatment session.

**Fig. 5:**
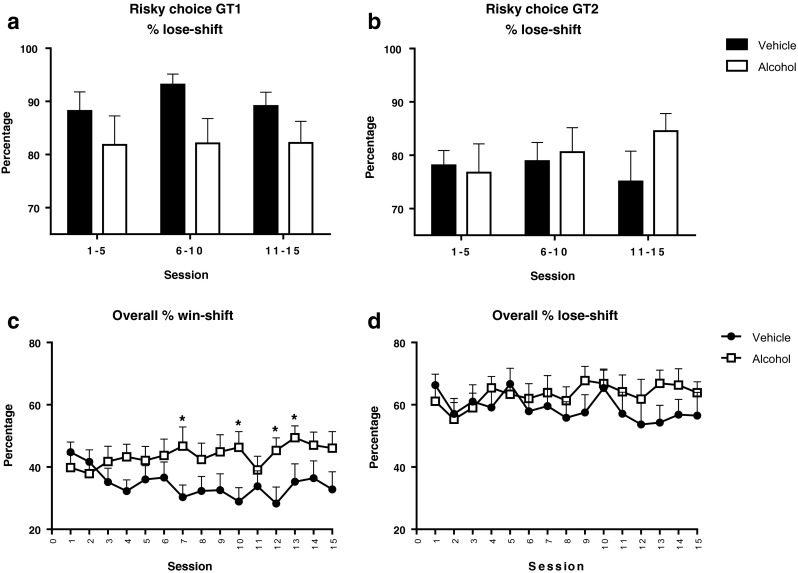
The effects of repeated alcohol (0.6 g/kg) or vehicle administration on the percentage of shifts toward another choice after being rewarded or punished. Repeated alcohol administration during rGT acquisition tended to decrease lose-shift behavior after punishment on the risky choice in GT1 (**a**), but not in GT2 (**b**). Regardless ofchoice or GT version, vehicle-treated animals showed reduced win-shift behavior over sessions, whereas alcohol-treated animals did not (**c**). The percentage of lose-shifts was not different over sessions or between treatment groups (**d**). Data are presented in bins of five sessions (**a**, **b**) or sessions (**c**, **d**) and are shown as the mean + SEM percentage of lose-shift and win-shift behavior. *Different from vehicle-treated animals (post hoc Student’s *t* test, *p* < 0.05)

The pattern of effects on choice behavior during alcohol treatment, in which alcohol increases risky choice only in the rGT version in which risky choice entailed greater punishment, suggests that alcohol reduces the ability to adjust behavior after punishment. To further investigate this interpretation, we performed trial-by-trial analyses of choice behavior to assess whether alcohol-treated animals respond differentially to positive (win) or negative feedback (loss), by shifting toward a different choice option on the next trial. We observed an interaction between treatment and rGT version in the percentage of lose-shifts following a risky choice (*F*_(1,28) treatment × GT_ = 4.37, *p* < 0.05) as well as a main effect of rGT version (*F*_(1,28) GT_ = 5.34, *p* < 0.05) (Fig. 5a, b). Subsequent analyses indicated that alcohol-treated animals in GT1 tended to perform less lose-shifts after punishment on the risky choice option (*F*_(1,14) treatment_ = 4.10, *p* = 0.062) (Fig. 5a), whereas alcohol did not affect lose-shift behavior in GT2 (*F*_(1,14) treatment_ = 0.88, NS) (Fig. 5b). The trial-by-trial analyses after feedback for the safe and optimal choice did not reveal significant differences between treatment groups or rGT version (data not shown). Analyses of the total percentage of shifts after a reward, regardless of which option was chosen, showed an interaction between treatment and session (*F*_(9,247) session × treatment_ = 2.69, *p* < 0.05), independent of rGT version (*F*_(9,247) session × treatment × GT_ = 0.76, NS), indicating that the vehicle-treated animals showed a reduction of win-shifts over sessions (*F*_(14,196) session_ = 2.95, *p* < 0.05), whereas the alcohol treated animals did not (*F*_(6,79) session_ = 0.95, NS) (Fig. 5c). Lose-shift behavior, i.e., percentage of shifts after a punishment, regardless of which option was chosen, was not altered by alcohol treatment (*F*_(12,340) session × treatment_ = 0.71, NS) (Fig. 5d).

Treatment with alcohol during rGT acquisition increased premature responding over sessions (*F*_(2,44) treatment × session_ = 3.55, *p* < 0.05), which was independent of rGT version (*F*_(2,44) treatment × session × GT_ = 1.15, NS) (Table 3). In addition, alcohol-treated animals made more perseverative responses during punishment trials compared to vehicle-treated animals (*F*_(1,28) treatment × GT_ = 6.05, *p* < 0.05), which was apparent in GT1 (*F*_(1,14) treatment_ = 6.86, *p* < 0.05) but not in GT2 (*F*_(1,14) treatment_ = 0.54, NS). After alcohol treatment was discontinued, alcohol-pretreated animals no longer differed from vehicle-pretreated animals in premature or perseverative responding (*F*_(1,28) treatment_ = 0.47, NS; *F*_(1,28) treatment_ = 1.12, NS, respectively). The numbers of choices and omissions were not different between treatment groups (*F*_(1,28) treatment_ = 0.69, NS; *F*_(1,28) treatment_ = 0.05, NS, respectively), but animals in GT1 made significantly more choices and less omissions compared to GT2, during alcohol treatment (*F*_(1,28) GT_ = 9.12, *p* < 0.05; *F*_(1,28) GT_ = 6.62, *p* < 0.05, respectively) and posttreatment (*F*_(1,28) GT_ = 3.58, *p* < 0.05; *F*_(1,28) GT_ = 3.26, *p* = 0.08, respectively). Choice latency declined over sessions during the treatment period (*F*_(2,56) session_ = 25.09, *p* < 0.05), independent of treatment group (*F*_(2,56) session × treatment_ = 1.32, NS) or rGT version (*F*_(2,56) session × GT_ = 0.29, NS). Collect latency changed over sessions during the treatment period, which was different for alcohol- and vehicle-treated animals as well as for the rGT versions (*F*_(2,54) treatment × GT × session_ = 5.50, *p* < 0.05). Post hoc analyses indicated that rats in GT1 were faster in their reward collection compared to rats in GT2 (*F*_(1,27) GT_ = 16.94, *p* < 0.05), which was independent from treatment (*F*_(1,27) treatment × GT_ = 0.00, NS). There were no differences in choice and reward collection latencies in the posttreatment period between the treatment groups (*F*_(1,28) treatment_ = 1.20, NS; *F*_(1,28) treatment_ = 0.52, NS, respectively) (Table 3).

**Table 3:** Effects of repeated treatment with 0.6 g/kg alcohol or vehicle during acquisition (15 sessions), followed by ten sessions without treatment on behavior in the rGT

	Treatment Sessions 1–15						Posttreatment Sessions 16–25				
Variable	Effect rGT	Effect Treatment	Treatment	1–5	6–10	11–15	Effect rGT	Effect Treatment	Treatment	1–5	6–10
Number of choices GT1	*F*_(1,28) rGT_ = 9.115, *p* < 0.05	*F*_(1,28) treatment_ = 0.690, NS	Vehicle	74.20 ± 5.50	77.78 ± 5.93	81.60 ± 5.64	*F*_(1,28) rGT_ = 5.577, *p* < 0.05	*F*_(1,28) treatment_ = 0.426, NS	Vehicle	78.00 ± 4.70	74.85 ± 5.99
									Alcohol	81.13 ± 3.25	81.52 ± 2.76
			Alcohol	74.73 ± 6.86	77.63 ± 4.77	74.73 ± 5.22					
Number of choices GT2			Vehicle	64.65 ± 4.19	68.25 ± 4.71	65.18 ± 4.95			Vehicle	78.41 ± 5.13	70.45 ± 3.9
									Alcohol	61.61 ± 4.30	65.15 ± 5.31
			Alcohol	62.55 ± 3.87	59.56 ± 4.03	57.58 ± 5.09					
Premature responses	*F*_(1,28) rGT_ = 2.014, NS	*F*_(2,45) treatment × session_ = 3.552, *p* < 0.05	Vehicle	11.71 ± 1.58	13.76 ± 1.50	12.89 ± 2.26	*F*_(1,28) rGT_ = 0.003, NS	*F*_(1,28) treatment_ = 0.472, NS	Vehicle	9.58 ± 1.36	10.73 ± 1.53
									Alcohol	11.46 ± 1.54	11.91 ± 1.59
			Alcohol	10.48 ± 1.67	13.61 ± 2.72	19.61 ± 3.85*					
PersevP GT1	*F*_(1,28) rGT_ = 20.825 *p* < 0.05	*F*_(1,14) treatment GT1_ = 6.864, *p* < 0.05	Vehicle	40.18 ± 7.48	26.23 ± 3.67	30.40 ± 5.06*	*F*_(1,28) rGT_ = 6.695, *p* < 0.05	*F*_(1,28) treatment_ = 2.415, NS	Vehicle	23.15 ± 3.21	23.38 ± 2.70
									Alcohol	33.00 ± 3.16	36.00 ± 4.38
			Alcohol	68.25 ± 18.30	59.25 ± 8.33^#^	41.13 ± 3.30					
PersevP GT2	*F*_(1,28) treatment × rGT_ = 6.052, *p* < 0.05	*F*_(1,14) treatment GT2_ = 0.537, NS	Vehicle	24.88 ± 4.47	20.50 ± 3.57	22.25 ± 4.70			Vehicle	17.25 ± 3.51	22.86 ± 3.06
									Alcohol	18.25 ± 3.39	23.88 ± 6.07
			Alcohol	24.50 ± 5.17	16.50 ± 3.22	16.25 ± 3.02					
Omissions GT1	*F*_(1,28) rGT_ = 6.624 *p* < 0.05	*F*_(1,28) treatment_ = 0.053, NS	Vehicle	18.80 ± 3.37	18.55 ± 3.51	14.33 ± 2.89	*F*_(1,28) rGT_ = 3.263 *p* = 0.082	*F*_(1,28) treatment_ = 0.217, NS	Vehicle	19.43 ± 3.66	20.08 ± 3.66
									Alcohol	17.18 ± 3.80	16.80 ± 3.62
			Alcohol	18.79 ± 7.14	12.13 ± 3.46	15.90 ± 4.57					
Omissions GT2			Vehicle	32.38 ± 6.69	24.28 ± 5.82	28.85 ± 4.82			Vehicle	25.05 ± 4.75	23.10 ± 5.13
									Alcohol	37.91 ± 6.60	31.43 ± 7.67
			Alcohol	26.18 ± 5.88	28.70 ± 5.12	36.13 ± 6.81					
Choice latency (sec)	*F*_(1,28) rGT_ = 2.482, NS	*F*_(1,28) treatment_ = 1.580, NS	Vehicle	3.49 ± 0.16	3.20 ± 0.17	3.10 ± 0.19	*F*_(1,28) rGT_ = 0.821, NS	*F*_(1,28) treatment_ = 1.204, NS	Vehicle	3.37 ± 0.19	3.35 ± 0.19
									Alcohol	3.09 ± 0.19	3.04 ± 0.23
			Alcohol	3.31 ± 0.20	2.90 ± 0.19	2.67 ± 0.18					
Collect latency (sec) GT1	*F*_(1,27) rGT_ = 16.942 *p* < 0.05	*F*_(2,28) treatment rGT1 × session_ = 2.891, *p* = 0.079	Vehicle	3.16 ± 0.33	2.58 ± 0.26	2.41 ± 0.24*	*F*_(1,28) rGT_ = 3.622, *p* = 0.067	*F*_(1,28) treatment_ = 0.517, NS	Vehicle	2.78 ± 0.40	2.57 ± 0.23
			Alcohol	2.74 ± 0.44	2.48 ± 0.28	1.80 ± 0.13					
									Alcohol	2.27 ± 0.40	2.40 ± 0.30
Collect latency (sec) GT2	*F*_(2,54) treatment × rGT × session_ = 5.500, *p* < 0.05	*F*_(2,26) treatment rGT2 × session_ = 2.733, *p* = 0.084	Vehicle	3.14 ± 0.38	3.75 ± 0.73	4.86 ± 0.94			Vehicle	2.61 ± 0.51	3.22 ± 0.68
									Alcohol	2.90 ± 0.35	4.12 ± 1.21
			Alcohol	4.00 ± 0.54	6.96 ± 2.63	5.19 ± 2.06					

Data are presented as means ± SEM in bins of five sessions during the treatment (sessions 1–15) and posttreatment period (sessions 16–25). Data from both rGT versions were pooled in case the rGT version did not interact with alcohol treatment (i.e., no GT × treatment interaction). Separate analyses were performed for the treatment and posttreatment period

*p < 0.05, different from the first session bin; #*p* < 0.05, different between treatments within the session bin

*PersevP* perseverative responses during a punishment trial, *NS* not significant

### Effects of alcohol challenges on rGT performance after alcohol treatment during rGT acquisition

**Fig. 6:**
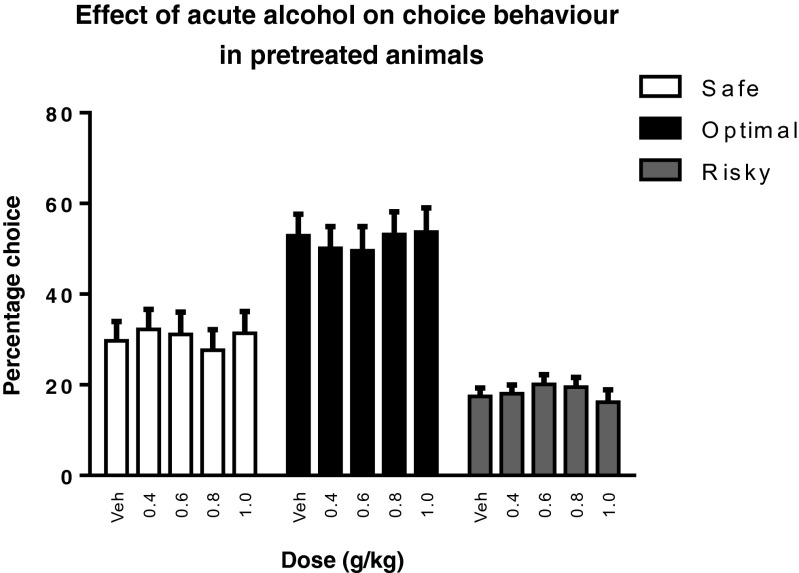
The effects of acute alcohol treatment on stable choice behavior in the rGT in experiment 2. Alcohol had no effects on choice behavior, irrespective of pretreatment (alcohol or vehicle) or rGT version. Hence, the data from both pretreatment groups and rGT versions were collapsed. Data are shown as the mean percentage choice + SEM

Subsequent to the rGT sessions without alcohol or vehicle treatment, the animals were challenged with alcohol (0.0–1.0 g/kg) to determine whether alcohol preexposure alters the effects of alcohol on rGT performance. Acute alcohol challenges did not change choice behavior (*F*_(7,190) dose × choice_ = 0.88, NS), irrespective of whether pretreatment group or rGT version were included as factors (*F*_(7,190) dose × choice × pretreatment_ = 0.54,NS; *F*_(7,190) dose × choice × GT_ = 0.93, NS, respectively) (Fig. 6).

**Fig. 7:**
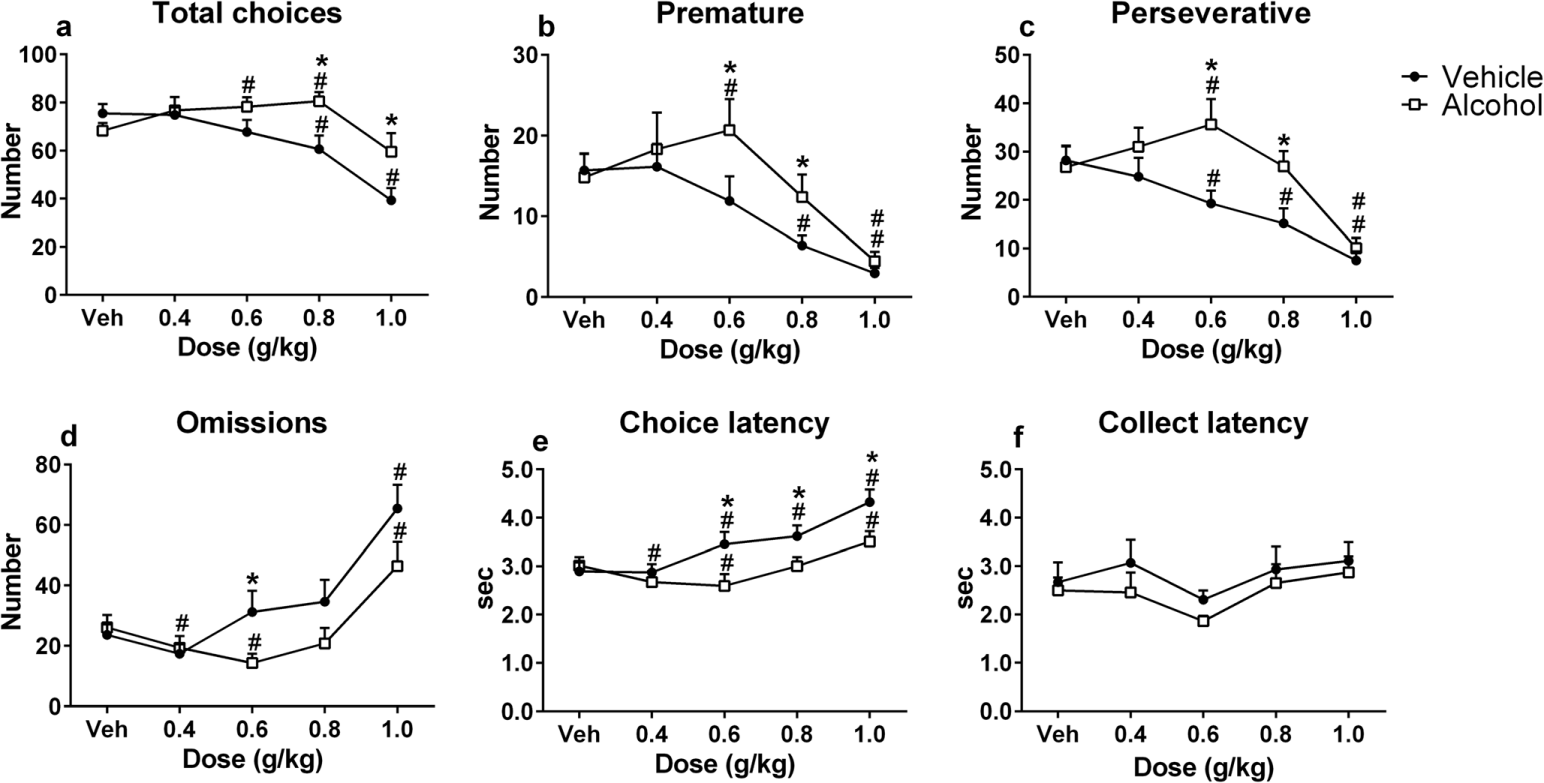
The effects of alcohol on behavior in the rGT during alcohol challenge sessions in alcohol-pretreated and vehicle-pretreated animals. In vehicle-pretreated animals, alcohol dose-dependently reduced total choices (**a**), premature responses (**b**), and perseverative responses (**c**), and increased omissions (**d**) and choice latency (**e**). In contrast, in alcohol-pretreated animals, alcohol had a biphasic effect on total choices, premature responses, perseverative responses (increase followed by decrease as the alcohol dose increased), omissions and choice latencies (decrease followed by increase as the alcohol dose increased). Alcohol pretreatment and alcohol challenges did not affect collect latency (**f**). The alcohol challenges had similar effects in GT1 and GT2, and the data were therefore pooled. Data are shown as mean + SEM. *Difference between pretreatment groups (post hoc Student’s *t* test, *p* < 0.05); #different from vehicle challenge (post hoc paired *t* test, *p* < 0.05)

Alcohol challenges differentially altered behavior in the rGT in alcohol- versus vehicle-pretreated animals (Fig. 7). In animals pretreated with alcohol, alcohol treatment had a biphasic effect, i.e., an increase followed by a decrease as the alcohol dose increased, on the total number of choices, premature and perseverative responses, whereas alcohol decreased these parameters in vehicle-pretreated animals (*F*_(4,120) dose × pretreatment_ = 4.94, *p* < 0.05; *F*_(3,105) dose × pretreatment_ = 2.70, *p* < 0.05; *F*_(4,109) dose × pretreatment_ = 3.76, *p* < 0.05, respectively) (Fig. 7a–c). Alcohol also had a biphasic effect, i.e., a decrease followed by an increase as the alcohol dose increased, on omissions and choice latency in alcohol-pretreated animals, whereas both were increased by alcohol in vehicle-pretreated animals (*F*_(3,96) dose × pretreatment_ = 3.14, *p* < 0.05; *F*_(4,120) dose × pretreatment_ = 4.24, *p* < 0.05, respectively) (Fig. 7d, e). Collect latency was not affected by alcohol challenges (*F*_(4,120) dose × pretreatment_ = 0.35, NS) (Fig. 7f).

## Discussion

We investigated the effects of alcohol on acquisition and performance in two versions of the rGT that differ in the net gain and the punishment magnitude associated with the different response options. In rats that showed stable rGT performance, alcohol modestly altered choice behavior and had behavioral suppressant effects on responding. In contrast, repeated alcohol administration during task acquisition enhanced risk taking in the rGT version in which risky choices entailed long punishment delays (i.e., GT1). Trial-by-trial analyses showed that alcohol-treated rats tended to show less lose-shift behavior after risky choices in GT1. Regardless of choice or rGT version, the decline in win-shift behavior during task acquisition was blunted in alcohol-treated rats. Furthermore, pretreatment with alcohol during rGT acquisition caused increases in premature and perseverative responding and a reduction in the behavioral suppressant effects of alcohol upon subsequent alcohol challenge. Together, these results suggest that repeated alcohol administration reduces the ability to use feedback to adjust choice behavior and unmasks its disinhibitory effects on behavior.

### Alcohol effects on rGT performance

Acute alcohol administration during baseline choice behavior in the rGT caused a small reduction in the percentage of optimal choices in experiment 1, but not in experiment 2, in which animals had been pretreated with alcohol or vehicle during task acquisition. Moreover, even if the vehicle-treated animals in experiment 2, which are more comparable to the alcohol-naive animals in experiment 1, were analyzed separately, no effect of the alcohol challenges on choice behavior was observed (data not shown). The slight inconsistency in the effect of alcohol on choice behavior between experiments 1 and 2 is not likely to result from procedural differences between the experiments. The animals in experiment 1 were subjected to five forced-choice sessions after five free-choice sessions, whereas the animals in experiment 2 directly received these five forced-choice sessions after animals reliably acquired nose-poke responding for food. Hence, the lack of these first five free-choice sessions preceding the forced-choice sessions might have influenced the acquisition curve in the second experiment. However, the number of training sessions that the animals received before alcohol challenges was comparable in the two experiments (i.e. five forced-choice sessions in both experiments and 26 free-choice sessions in experiment 1 versus 25 free-choice sessions in experiment 2). Importantly, the effects of acute alcohol exposure on other rGT parameters were consistent between both experiments. Together, these results suggest that alcohol has modest effects on established decision making in the rGT.

These findings are in agreement with a recent report (Peña-Oliver et al. 2014) that showed no effect of alcohol on decision making in a mouse version of the IGT. Moreover, in a task where rats were trained to choose between a small food reward and a large food reward that was accompanied by probabilistic footshock, alcohol did not affect choice behavior (Mitchell et al. 2011). However, the alcohol doses tested here did affect behavior in the rGT, as they induced decreases in the number of choices, premature and perseverative responses, and increased omissions and latencies, indicating that alcohol has behavioral suppressant effects in the rGT, even if choice behavior itself is hardly affected.

Human studies on the effects of alcohol on choice behavior have yielded mixed results. For example, healthy participants showed more risky decision making under the influence of alcohol (Lane et al. 2004; George et al. 2005) but negative results have also been reported (Ramaekers and Kuypers 2006). Of note, in the rGT, animals are typically trained for several weeks until they have established a stable choice pattern and the animals have learned which option results in the highest gain before pharmacological challenges are performed. As a result, the animals have developed a stable choice strategy, which apparently is quite insensitive to alcohol. However, stable choice behavior in the rGT has previously been shown to be sensitive to pharmacological manipulations, although in most cases other parameters such as premature responses are influenced as well (Zeeb et al. 2009; Baarendse et al. 2013). In human studies, the IGT is performed in one single session, both under uncertain, i.e., when the task contingencies are not fully known, and more certain conditions, i.e., when the choice contingencies become known to the participant. Hence, alcohol treatment during acquisition of the rGT may be more comparable to acute alcohol treatment in the human IGT. That said, because the rGT requires a certain number of training sessions, the animals will receive multiple alcohol treatments, whereas a human study only requires a single treatment with alcohol. Hence, the repeated versus single alcohol administrations might result in different effects on choice behavior. Moreover, it is likely that different types of memory processes are used within a single session and between sessions. In the human situation, IGT performance relies on working memory processes, whereas in the rGT both working memory during the session as well as long-term memory between sessions contribute to the animals’ choice behavior. Therefore, we cannot rule out that differential effects of alcohol on these types of memory cause divergent effects in the human IGT and the rGT.

### Alcohol effects on rGT acquisition

We found enhanced risky choice behavior in animals treated with alcohol during task acquisition, selectively in GT1. This indicates that under uncertain conditions, the effects of alcohol on decision making are more pronounced, albeit that they are dependent on the structure of the task. Subsequent to the alcohol treatment sessions, animals were tested for another ten sessions without treatment. In these sessions, there was no difference in choice behavior between alcohol- and vehicle-pretreated animals. Thus, although alcohol affected decision making during treatment, it had no lasting consequences for choice behavior. The most important difference in contingencies for the risky choice option between the two rGT versions is the length of the punishment timeout, which is four times longer in GT1 than in GT2. Whereas vehicle-treated animals showed a gradual reduction in risky choices over test sessions in GT1, the alcohol-treated animals took much longer to adapt their behavior after punishment after a risky choice. Trial-by-trial analysis revealed that the alcohol-treated animals in GT1 tended to show less lose-shift behavior after being punished following a risky choice. This was not observed in GT2. Importantly, in both GT1 and GT2, the risky choice provided the animal with a probabilistic large reward. Hence, if alcohol promotes risky behavior, then risky decision making should have been increased in both rGT versions. In contrast, if alcohol increases reward sensitivity, it is expected that animals make more risky choices in GT2, since the reward magnitude after risky choices is higher in GT2 (six pellets) than in GT1 (four pellets). Together, these results suggest that alcohol-induced risky behavior results from an impaired capacity to adapt choice behavior on the basis of negative feedback, rather than making animals more risk-prone, or alter their reward sensitivity.

During rGT acquisition, vehicle-treated animals showed a steady decline in win-shift behavior, i.e., less shifts toward another choice option after being rewarded, irrespective of which option was rewarded on the previous trial and irrespective of rGT version. Theoretically, it is expected that the number of shifts after being rewarded declines over sessions as the animals gradually come to show more exploitative decision making over sessions, as has been shown in human IGT studies (Bechara et al. 1994, 2001). Interestingly, alcohol treatment blunted this decline in win-shift behavior, suggesting that the alcohol-treated animals are impaired in adjusting their behavior upon feedback, resulting in reduced shifting from an explorative towards an exploitative decision-making strategy.

Our findings are in line with human studies, in which AUD patients need more trials to shift toward advantageous choices in the IGT (Kim et al. 2011), which may be caused by a reduced sensitivity to losses and a bias towards trials with gains (Gullo and Stieger 2011). Studies using other risky decision making tasks show similar findings, in that AUD patients fail to adjust their behavior after experiencing negative consequences in the Balloon Analogue Risk Task (Holmes et al. 2009) and the monetary Go/No-Go task (Rossiter et al. 2012). However, comparison of these studies with our data has to be done with caution, as we investigated the acute effect of alcohol on rGT performance and not the long-term effects of alcohol abuse. Nevertheless, studies on acute alcohol exposure in healthy individuals describe comparable findings (George et al. 2005; Loeber and Duka 2009a, b), suggesting that alcohol disrupts the ability to alter behavior after negative feedback.

Repeated alcohol administration also increased premature responses over sessions (Table 3), showing that repeated alcohol administration caused disinhibitory effects on behavior. However, these effects on impulsive behavior occurred later (i.e., sessions 11–15) than the effects of alcohol on risky choice (which was apparent from session 6 onward), and in both rGT versions. This indicates that repeated alcohol treatment results in impulsive behavior, but likely through a different mechanism than its effects on decision making. Alcohol treatment may also have affected time perception, so that the long punishment time-out after a risky choice was not perceived as such. Importantly, however, preclinical studies on impulsive choice in delay discounting tasks have shown that alcohol increases choice for a small immediate reward (Tomie et al. 1998; Evenden and Ryan 1999; Olmstead et al. 2006; Wilhelm and Mitchell 2012). Thus, if alcohol alters time perception in a way that a long delay or time-out period is not perceived as such, one would expect alcohol to increase, and not decrease choice for the large delayed reward. Interestingly, in a recent detailed analysis of delay discounting in rats, alcohol did not affect the sensitivity to delay or reward size (Moschak and Mitchell 2013). Moreover, human studies have not shown consistent acute alcohol effects on delay discounting (Richards et al. 1999; Ortner et al. 2003; Reynolds et al. 2006; Bidwell et al. 2013). We therefore consider it unlikely that alcohol influenced choice behavior in the rGT as a result of altered time perception.

### Differential effects of alcohol treatment in alcohol-pretreated animals and controls

We observed no effect of acute alcohol challenges on decision making in experiment 2, irrespective of whether animals were pretreated with alcohol or vehicle during rGT acquisition. Hence, previous alcohol treatment did not alter the acute effects of alcohol on choice behavior. However, animals pretreated with alcohol exhibited behavioral disinhibition upon treatment with low to moderate doses of alcohol, where these doses increased the number of total choices, premature and perseverative responses and decreased omissions and choice latencies. In contrast, vehicle-pretreated animals showed dose-dependent behavioral suppressant effects of alcohol, similar to the acute alcohol challenges in experiment 1. Thus, behavioral disinhibition in rodents may be unmasked after pretreatment with alcohol, alongside with tolerance to its suppressant effects. The biphasic dose effect of alcohol, where moderate doses induce disinhibition and high doses predominantly result in sedation in both human and animals, is a well-known characteristic of alcohol (Pohorecky 1977). We observed similar biphasic effects of alcohol in alcohol-pretreated animals, but in vehicle-treated animals, alcohol had merely behavioral suppressant effects. Previous five-choice serial reaction time task (5CSRTT) studies have shown that motor impulsivity upon alcohol challenge only increased after multiple cycles of alcohol intoxication and abstinence (Walker et al. 2011; Irimia et al. 2013). In contrast, acute alcohol challenges in otherwise alcohol-naive animals did not affect impulsive action (Peña-Oliver et al. 2009; Semenova 2012) or reduced impulsivity in the 5CSRTT (Bizarro et al. 2003), the latter being consistent with our findings. Other preclinical studies found that alcohol increases motor impulsivity in naive animals, but only when a novelty component is introduced (Peña-Oliver et al. 2009; Walker et al. 2011; Irimia et al. 2013). In agreement with the present findings, acute alcohol administration resulted in tolerance to the sedative effects of acute alcohol in adult rats that were pretreated with alcohol during adolescence (Matthews et al. 2008; Semenova 2012). These findings are reminiscent of findings in humans, where heavy alcohol users act more impulsively and report feeling more stimulated after alcohol exposure compared to light users (Marczinski et al. 2007; King et al. 2011; Reed et al. 2012), which may impair the ability to refrain from drinking. Moreover, people who recently consumed alcohol display less alcohol-induced impairments in motor coordination (Miller et al. 2012). Taken together, acute alcohol challenges after a period of abstinence from repeated alcohol treatment result in disinhibition of behavior.

## Concluding remarks

The experiments described in this study demonstrate augmented risk taking behavior after alcohol treatment during conditions of uncertainty, e.g., during task acquisition. This may be due to a reduced ability to adjust choice behavior on the basis of feedback. Moreover, alcohol preexposure unmasks its disinhibitory effects on behavior. Impaired responsiveness to choice feedback and behavioral disinhibition may therefore contribute to the development of AUD.
